# Effect of Nordic Walking on Anthropometrics, Glycemia, and Lipid Profile in Adults With Prediabetes or Diabetes: A Systematic Review and Meta‐Analysis of Randomized Controlled Trials

**DOI:** 10.1155/jdr/5886930

**Published:** 2026-01-08

**Authors:** Sichao Chen, Xiaohong An, Ankang Wu, Yubo Liu, Veeranjaneya Reddy Lebaka, Bhaskar LVKS, Mallikarjuna Korivi, Weibing Ye

**Affiliations:** ^1^ Institute of Human Movement and Sports Engineering, College of Physical Education and Health Sciences, Zhejiang Normal University, Jinhua, Zhejiang, China, zjnu.edu.cn; ^2^ College of Physical Education and Health, Yili Normal University, Yining, Xinjiang, China, ylsy.edu.cn; ^3^ Department of Microbiology, Yogi Vemana University, Kadapa, Andhra Pradesh, India, yogivemanauniversity.ac.in; ^4^ Department of Zoology, Guru Ghasidas Vishwavidyalaya, Bilaspur, Chhattisgarh, India, ggu.ac.in

**Keywords:** blood pressure, body weight, cholesterol, HbA1c, hyperglycemia, pole walking

## Abstract

**Background:**

Lifestyle interventions, including Nordic walking, a whole‐body exercise session, are beneficial for metabolic diseases, including diabetes. However, evidence from research studies or meta‐analyses on the efficacy of Nordic walking in improving clinical outcomes in patients with diabetes/prediabetes remains inconclusive.

**Purpose:**

This systematic review and meta‐analysis investigated the efficiency of Nordic walking on anthropometrics, glycemic control, lipid profiles, blood pressure, and peak oxygen uptake (VO_2_peak) in adults with prediabetes/diabetes.

**Methods:**

A systematic literature search was performed across PubMed, Web of Science, the Cochrane Library, Scopus, and Embase until June 2025. We included randomized controlled trials (RCTs) involving adults with prediabetes or diabetes that compared the Nordic walking effect with a control trial. Data on clinical outcomes, including anthropometrics, glycemic control, lipid profiles, blood pressure, and VO_2_peak, were extracted for meta‐analysis and presented as mean difference (MD).

**Results:**

A total of six RCTs, comprising 321 adults with prediabetes or diabetes (63% male), were included in the analysis. Meta‐analysis results showed that compared with control, Nordic walking significantly decreased body weight (MD = −0.79 kg, *p* = 0.02), and marginally reduced waist circumference (MD = −0.82 cm, *p* = 0.07). Notably, Nordic walking decreased glycated hemoglobin (HbA1c) (MD = −0.37*%*, *p* = 0.0001) but showed high heterogeneity (*I* ^2^ = 69*%*). Subgroup analysis revealed a greater reduction of HbA1c in adults with diabetes (MD = −0.49*%*, *p* < 0.00001) than that of adults with prediabetes (MD = −0.2*%*, *p* = 0.001). However, Nordic walking did not affect fasting blood glucose or homeostasis model assessment of insulin resistance (HOMA‐IR). Additionally, Nordic walking significantly increased high‐density lipoprotein (HDL) cholesterol (MD = 0.07 mmol/L, *p* = 0.005), while showing no beneficial effects on total cholesterol, triglycerides, and low‐density lipoprotein. Nordic walking appears to improve VO_2_peak but does not affect systolic or diastolic blood pressure.

**Conclusions:**

Nordic walking exerted beneficial effects on body weight, HbA1c, and HDL in adults with prediabetes or diabetes. Our findings support that Nordic walking can be a practical intervention in managing or treating diabetic complications.

## 1. Introduction

Type 2 diabetes is characterized by elevated blood glucose levels or higher concentrations of glycated hemoglobin (HbA1c). The higher HbA1c level, ranging from 5.7% to 6.4%, is defined as impaired glucose tolerance (IGT) or prediabetes, which progresses to Type 2 diabetes if not controlled [1, 2]. Individuals with prediabetes or Type 2 diabetes have a greater risk of developing cardiovascular diseases (CVDs) compared with those with normoglycemia [3, 4]. According to the latest data from the International Diabetes Federation (IDF), there are 634.8 million adults with prediabetes and 588.7 million adults with diabetes living worldwide. The number of prediabetes and diabetes cases is projected to increase to 846.5 and 852.5 million, respectively, by 2050 [5]. Unhealthy lifestyle behaviors, including physical inactivity, prolonged sedentary time, and poor dietary habits, can alter anthropometrics and glycemic levels, which may progress to prediabetes or diabetes over time [6–9]. Practicing a healthy lifestyle includes engaging in regular exercise programs, either aerobic or resistance training, which is strongly associated with improved glycemic control in people with prediabetes or diabetes [10–12]. Aerobic exercise is an effective intervention that decreases body weight, HbA1c, and hyperlipidemia in patients with diabetes [13–15].

Nordic walking, a form of aerobic exercise, was developed in Scandinavia and is a popular practice in Europe [16]. Nordic walking is a specialized form of exercise like skiing that incorporates specially designed poles to combine upper body movement with walking and allows for greater energy expenditure. Approximately 70%–90% of the body′s skeletal muscles are involved during Nordic walking [17]. Compared with normal walking without poles, Nordic walking with poles can increase walking distance in patients with intermittent claudication [18]. Nordic walking can be incorporated into the daily exercise routine and is claimed to enhance aerobic capacity, strength, and quality of life of older adults [19]. Existing results from randomized control trials (RCTs) showed inconclusive effects of Nordic walking on glycemic control and lipid profiles in people with prediabetes or diabetes. For instance, one RCT conducted on overweight/obese adults with diabetes or prediabetes (body mass index [BMI] > 30 kg/m^2^, age above 59 years) demonstrated that 16‐week unsupervised Nordic walking decreased HbA1c in adults with diabetes but not in adults with prediabetes, whereas the lipid profile remained unchanged in both groups [20]. In contrast, another RCT recruited overweight/obese adults with prediabetes (mean BMI: 30 kg/m^2^, mean age: 55 years) and showed that 12‐week Nordic walking (supervised) did not affect blood glucose, HbA1c, and high‐density lipoprotein (HDL) cholesterol compared with control [21]. Furthermore, 6‐month supervised Nordic walking significantly improved fasting blood glucose (FBG) and systolic blood pressure (SBP) in older adults with diabetes (mean BMI 27 kg/m^2^, age 65 years) but did not affect HbA1c, total cholesterol (TC), triglycerides (TG), and low‐density lipoprotein (LDL) levels compared with baseline [22].

Systematic reviews and meta‐analyses represent the highest levels of clinical evidence and are fundamental in developing clinical practice guidelines [23]. Some systematic reviews and meta‐analyses have reported the clinical significance of Nordic walking for various populations, including those with CVDs [17, 24, 25], respiratory diseases [26], Parkinson′s disease [27], and overweight/obesity [28]. However, no meta‐analysis has synthesized the evidence to address the effect of Nordic walking on clinical outcomes in adults with prediabetes or diabetes. Although few meta‐analyses explored the impact of Nordic walking on glycemic control or lipid profile, the included patients in those meta‐analyses were not exclusively diabetic, and the synthesized evidence showed diverse effects. One meta‐analysis of adults with overweight or obesity (eight RCTs, *n* = 465) found no significant benefits of Nordic walking on HbA1c, homeostasis model assessment of insulin resistance (HOMA‐IR), BMI, and lipid profiles compared with control [28]. A recent meta‐analysis of 22 RCTs comprising 1,271 older adults (mean age 62 years) concluded that Nordic walking had no effect on HbA1c but improved other cardiovascular risk factors, such as BMI, TC, TG, and SBP [24]. These conflicting results from RCTs [20–22] and meta‐analyses [24, 28] can be attributed to certain limitations, including broader inclusion criteria, no restriction on health status, overlooking of baseline glycemic levels, significant demographic heterogeneity, and relatively smaller sample size. This evidence gap is critical because prediabetes is strongly associated with increased risk of CVDs and all‐cause mortality [4, 29]. An intervention that effectively manages prediabetes not only prevents the incidence of diabetes but also decreases the risk of developing CVDs. Therefore, we conducted this systematic review and meta‐analysis focusing exclusively on adults with prediabetes and diabetes to determine the efficacy of Nordic walking. Unlike previous meta‐analyses with broader inclusion criteria, our meta‐analysis included RCTs of adults with prediabetes or diabetes, and it is the first study to investigate the effects of Nordic walking on both prediabetes and diabetes groups via subgroup analysis. We examined the effects of Nordic walking on anthropometric measures, glycemic control, lipid profiles, and blood pressure.

## 2. Methods

### 2.1. Protocol Registration and Search Strategy

This systematic review was registered in PROSPERO with the registration number CRD420251024310. We conducted a literature search on PubMed, Web of Science, the Cochrane, Scopus, and Embase from inception until June 2025. A comprehensive search strategy for primary studies was developed and performed by three authors (S.C., X.A., and A.W.). The specific keywords used in the search are as follows: “Nordic walking” OR “walking Nordic” OR “Nordic exercise” OR “pole walking” OR “walking pole” AND “Type 2 diabetes mellitus” OR “Type 2 diabetes” OR “diabetes” OR “T2DM” OR “prediabetes” OR “insulin resistance” OR “impaired glucose tolerance” OR “homeostasis model assessment of insulin resistance” OR “HOMA‐IR”. The search was performed to find RCTs. Alternatively, a manual search was also conducted from the reference list of all included articles for any relevant studies to be included. The article search and review process was conducted strictly in accordance with the latest guidelines from the Preferred Reporting Items for Systematic Reviews and Meta‐Analysis (PRISMA) [30]. Further details are given in the PRISMA checklist, and the checklist is provided as Table S1. The complete search strategy on each database with the keywords is provided in Table S2.

### 2.2. Inclusion and Exclusion Criteria

As we aimed to explore the effect of Nordic walking on health‐related outcomes in individuals with diabetes or prediabetes, we adopted the following inclusion and exclusion criteria for the study. The inclusion criteria were (1) all participants must be adults with prediabetes or Type 2 diabetes; (2) the study should be an RCT with Nordic walking and a control trial; (3) the study should report at least one of the diabetes‐related markers; (4) the duration of the trial intervention should be 4 weeks or more; and (5) studies should be published in English as full‐text articles. The exclusion criteria were (1) participants with Type 1 diabetes; (2) Nordic walking combined with other interventions; and (3) the study did not report the relevant outcome indicators or had insufficient data. According to the above‐mentioned criteria, two authors (S.C. and A.W.) independently conducted a thorough review and evaluation of the relevant articles. Any differences in opinion on the inclusion or exclusion of articles were resolved by discussing with the corresponding authors (M.K. and W.Y.).

### 2.3. Outcome Measures and Data Extraction

Three authors (S.C., X.A., and A.W.) independently extracted the study details and outcome measures data of all included articles. The other authors (Y.L., V.R.L., and B.LVKS) verified the data. Any discrepancies were resolved by other authors (M.K. and W.Y.) through discussion. The information extracted from each article includes: study details (authors, publication year, and region where the study was conducted), participants′ characteristics (sample size, mean age, and gender), intervention details (frequency and duration), and a list of outcome measures (body composition, diabetes markers, lipid profile, blood pressure, and VO_2_peak). Specific details of all extracted data were presented in Table 1.

**Table 1:** Characteristics of the included studies.

**Study details**	**Participants′ characteristics**					**Intervention**		**Outcomes**
**Author, year country**	**Sample size (M/F)**	**Age (years)** **m** **e** **a** **n** ± **S** **D**	**HbA1c (%)** **m** **e** **a** **n** ± **S** **D**	**BMI (kg/m** ^**2**^ **)** **m** **e** **a** **n** ± **S** **D**	**TC, TG (mmol/L)** **m** **e** **a** **n** ± **S** **D**	**Time/frequency**	**Duration**	
Athwale and Shukla, 2024 [31] India	Con: 35 (20/15) T2D: 35 (17/18)	Con: 51.2 ± 7.6 T2D: 49.6 ± 7.2	Con: 7.5 ± 1.4 T2D: 8.0 ± 1.2	Con: 22.9 ± 2.2 T2D: 22.7 ± 1.9	NR	60 min/session 5‐time/week	4 weeks	HbA1c, VO_2_max, distance walked
Fritz et al. 2013 [20] Sweden	Con: 21 (10/11) IGT: 14 (5/9) Con: 30 (20/10) T2D: 20 (13/7)	Con: 61.8 ± 3.4 IGT: 59.1 ± 6.2 Con: 61.0 ± 4.7 T2D: 61.4 ± 4.6	Con: 5.9 ± 0.3 IGT: 5.9 ± 0.4 Con: 6.9 ± 0.9 T2D: 7.1 ± 0.9	Con: 30.8 ± 3.5 IGT: 32.0 ± 5.2 Con: 31.1 ± 3.9 T2D: 31.7 ± 5.2	Con: 6.0 ± 0.9 Con: 1.7 ± 0.9 IGT: 5.5 ± 0.9 IGT: 1.7 ± 1.2 Con: 4.5 ± 0.8 Con: 1.3 ± 0.4 T2D: 4.8 ± 0.9 T2D: 1.7 ± 0.7	5 h/week	16 weeks	Body weight, BMI, SBP, DBP, waist circumference, HbA1c, HOMA‐IR, p‐glucose fasting, p‐glucose 2 h post‐load, TG, TC, HDL, LDL, VO_2peak_
Gram et al. 2010 [32] Denmark	Con:22 (13/9) T2D:22 (10/12)	Con: 61.0 ± 10.0 T2D: 62.0 ± 10.0	Con: 7.8 ± 1.3 T2D: 7.2 ± 1.0	Con: 32.8 ± 4.0 T2D: 31.4 ± 4.3	Con: 4.6 ± 1.4 Con: NR T2D: 4.3 ± 0.9 T2D: NR	45 min/session 2‐time/week for first 8 weeks 1‐time/week for final 8 weeks	16 weeks	HbA1c, body weight, BMI, VO_2_max, waist circumference, SBP, DBP, hip circumference, TC, HDL, LDL
Jabardo‐Camprubí et al. 2023 [33] Spain	Con: 12 (7/5) T2D: 11 (8/3)	Con: 72.1 ± 10.0 T2D: 67.7 ± 5.1	Con: 6.7 ± 1.5 T2D: 7.3 ± 2.3	Con: 31.2 ± 8.0 T2D: 31.9 ± 6.2	NR	2‐time/week	12 weeks	Waist circumference, HbA1c, BMI
Sentinelli et al. 2015 [34] Italy	Con: 10 (0/10) T2D: 10 (0/10)	Con: 60.0 ± 5.0 T2D: 54.0 ± 9.0	Con: 7.1 ± 1.3 T2D: 7.1 ± 1.3	Con: 32.3 ± 6.0 T2D: 32.0 ± 7.0	Con: 5.2 ± 1.1 Con: 1.2 ± 0.7 T2D: 4.3 ± 0.8 T2D: 1.4 ± 0.5	60‐90 min/session 3‐time/week	12 weeks	Body weight, BMI, TC, TG, HDL, LDL, fasting blood glucose
Venojärvi et al. 2012 [21] Finland	Con: 40 (40/0) IGT: 39 (39/0)	Con: 54.0 ± 7.2 IGT: 55.0 ± 6.2	Con: 5.4 ± 0.6 IGT: 5.5 ± 0.6	Con: 28.6 ± 3.0 IGT: 30.0 ± 3.4	Con: 5.2 ± 1.3 Con: 1.6 ± 1.3 T2D: 5.3 ± 1.3 T2D: 1.9 ± 1.3	60 min/session 3‐time/week	12 weeks	HbA1c, TC, TG, HDL, LDL, HOMA‐IR, insulin, 2‐h insulin, glucose, 2‐h glucose

Abbreviation: M, male; F, female; Con, control; T2D, Type 2 diabetes; IGT, impaired glucose tolerance; HbA1c, glycated hemoglobin; VO_2_max, maximal oxygen consumption; VO_2_peak, peak oxygen uptake; BMI, body mass index; SBP, systolic blood pressure; DBP, diastolic blood pressure; TC, total cholesterol; TG, triglyceride; HDL, high‐density lipoprotein; LDL, low‐density lipoprotein; HOMA‐IR, homeostatic model assessment of insulin resistance; NR, not reported.

### 2.4. Quality Assessment

The quality of the included RCTs was assessed using the Cochrane risk of bias tool, version 2.0 (RoB 2) [35]. The RoB 2 consists of five domains (1) bias arising from the randomization process; (2) bias due to deviations from intended interventions; (3) bias due to missing outcome data; (4) bias in measurement of the outcome; and (5) bias in selection of the reported result. The quality of each domain was rated as “low risk of bias,” “high risk of bias,” or “some concerns,” and labelled with green (+), red (−), or yellow (!), respectively. The quality of the trials was assessed by the two authors (S.C. and A.W.). Any discrepancies were resolved through discussion with a third investigator (Y.L. and M.K.).

### 2.5. Statistical Analysis

We used Excel and RevMan 5.3 (Review Manager, the Cochrane collaboration) for data aggregation and analysis. One author was responsible for data collection, and another author was responsible for checking the extracted data. Disagreements were resolved by consensus decision. The effect sizes between the Nordic walking and the control groups were compared using the mean differences and 95% confidence intervals (CIs), and a forest plot was generated. Statistical differences were considered significant when the *p* value was less than 0.05. Heterogeneity was assessed using the *I* ^2^ statistic. An *I* ^2^ value of less than 50% indicates low heterogeneity, and a fixed‐effects model was employed. If the *I* ^2^ value is greater than 50%, it indicates high heterogeneity, and a random‐effects model was employed. Subgroup analysis was conducted to explore the sources of heterogeneity if the heterogeneity value is more than 50%. Statistical power for each outcome was reported using R (version 4.4.3). Sensitivity analysis was performed using the “leave‐one‐study‐out” method to evaluate the robustness of the pooled effect estimates for all outcomes. In this analysis, we systematically removed one trial at a time and reanalyzed the data to observe the impact on overall significance.

## 3. Results

### 3.1. Qualitative Analysis

#### 3.1.1. Summary of Search Results

**Figure 1:**
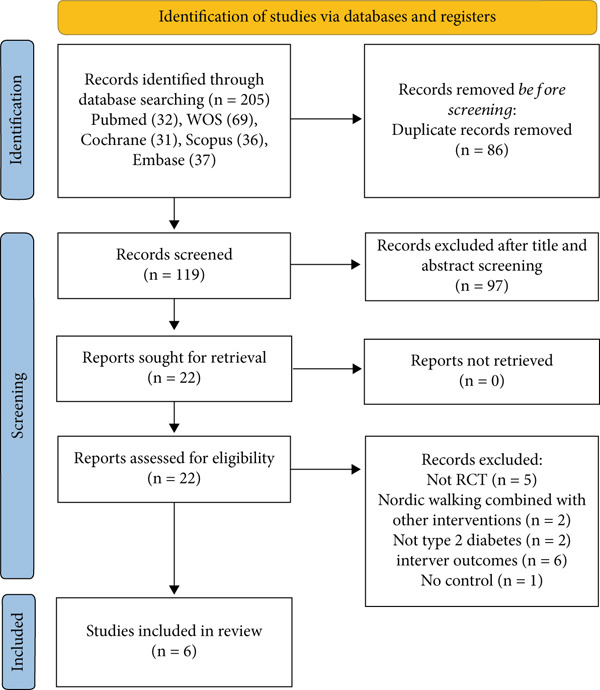
PRISMA flow chart.

Initially, we identified 205 studies through electronic databases, which include 32 from PubMed, 69 from Web of Science, 31 from the Cochrane, 36 from Scopus, and 37 from Embase. After removing the 86 duplicates, 119 studies remained. By reviewing the titles and abstracts, we excluded 97 studies, leaving 22 potential studies for inclusion. We then carefully reviewed the full text of the 22 studies and excluded 16 for the following reasons: five studies are not RCTs [22, 36–39], two studies employed Nordic walking with other interventions [40, 41], two studies are not on patients with Type 2 diabetes [42, 43], six studies reported no relevant outcomes [44–49], and one study had no comparable control group [50]. Finally, six studies met the inclusion criteria and were included in the systematic review and meta‐analysis. The number of studies in each step was clearly presented in the PRISMA flow chart (Figure 1).

#### 3.1.2. Characteristics of Included Studies and Participants

The included six RCTs comprising seven trials were published between 2010 and 2024. Five RCTs are from Europe, and one is from India. All trials investigated the effects of Nordic walking on individuals with prediabetes or diabetes, aiming to improve their health‐related outcomes. Among the six RCTs, five studies were designed as double‐arm RCTs [21, 31–34], and one study was designed as a multi‐arm RCT [20]. Overall, a total of 321 participants were enrolled in these studies, with 63% being male (male = 202, female = 119). The participants with prediabetes were 114, and the participants with diabetes were 207. The mean age of the participants was 58.63 years. Four studies were conducted on people with diabetes [31–34], one study conducted on both people with prediabetes and diabetes (independently) [20], and one study recruited only people with prediabetes [21]. The disease duration in patients with diabetes is more than 1 year. The Nordic walking intervention duration was 16‐week in two studies [20, 32] 12‐week in three studies [21, 33, 34], and 4‐week in one study [31]. Nordic walking session times ranged from 45‐ to 90‐min. Further characteristics of participants (baseline) and intervention protocol are presented in Table 1.

#### 3.1.3. Risk of Bias Assessment

The risk of bias for the included studies was evaluated using the Cochrane risk of bias tool Version 2.0 (RoB 2) [35], and the results are summarized in Figure S1. Four of the included studies were assessed to have a low risk of bias for the “randomization process” [20, 21, 31, 33]. Although other studies reported the use of randomization, they did not provide sufficient methodological details, which may introduce a risk of selection bias. The included RCTs are judged to have either some concerns or a high risk of bias in the domain of “deviations from intended interventions.” All studies were judged to have a low risk of bias in the domains “missing outcome data” and “measurement of the outcome.” Four studies were assessed as having some concerns regarding the “selection of the reported results” [20, 31, 32, 34]. In general, the included RCTs exhibited a relatively low risk of bias for the “overall bias.”

### 3.2. Quantitative Analysis

#### 3.2.1. Nordic Walking Decreases Body Weight, Not BMI and WC

The effect of Nordic walking on anthropometric variables in participants was assessed by meta‐analysis, and the results are presented in Figure 2. Changes in body weight were reported in four trials [20, 21, 32, 34], and the differences were compared between Nordic walking and control trials. The meta‐analysis results showed a significant reduction in body weight of participants following Nordic walking (Figure 2a; MD = −0.79 kg, 95% CI: −1.47 to −0.12, *I* ^2^ = 13*%*, *p* = 0.02, power = 80.5*%*). The changes in BMI were reported in four trials [20, 32–34], and we found no significant differences between Nordic walking and control trials (Figure 2b; MD = −0.10 kg/m^2^, 95% CI: −0.45 to 0.25, *I* ^2^ = 0*%*, *p* = 0.58, power = 8.5*%*). Next, we analyzed waist circumference (WC) data and found Nordic walking tended to decrease WC in participants (Figure 2c; MD = −0.82 cm, 95% CI: −1.71 to 0.06, *I* ^2^ = 0*%*, *p* = 0.07, power = 44.8*%*). Our meta‐analysis results indicate that Nordic walking produced a moderate but statistically significant weight reduction but was not effective in decreasing the BMI and WC of participants with prediabetes or diabetes. Furthermore, we calculated the percentage of weight loss for each study using baseline and postintervention values. We noticed that the percentage of weight loss in adults with prediabetes or diabetes is highly variable (0.5%–2.9%) across trials. This heterogeneity and smaller sample size may be attributed to the moderate magnitude of weight loss in our study (Table S3).

Figure 2Forest plot demonstrating the efficacy of Nordic walking on anthropometrics in adults with prediabetes or Type 2 diabetes. (a) Body weight; (b) body mass index (BMI); and (c) waist circumference (WC). SD, standard deviation; CI, confidence interval; IGT, impaired glucose tolerance; T2D, Type 2 diabetes.

**Figure:**
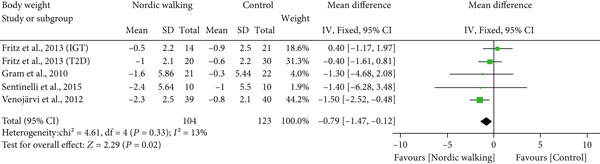
(a)

**Figure:**
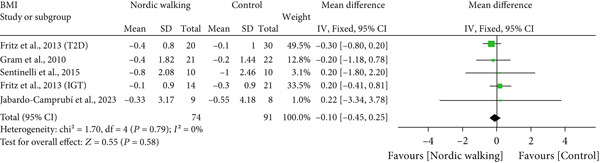
(b)

**Figure:**
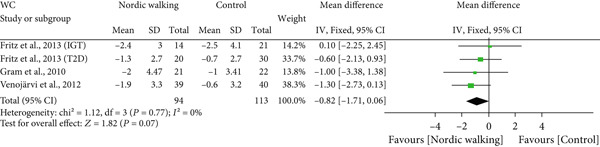
(c)

#### 3.2.2. Nordic Walking Decreases HbA1c

All included studies reported at least one diabetes indicator. To be specific, five studies (six trials) reported HbA1c [20, 21, 31–33], three studies (four trials) reported FBG [20, 21, 34], two studies (three trials) reported 2‐h glucose [20, 21], and two studies (three trials) reported HOMA‐IR [20, 21] data. The pooled meta‐analysis results showed that Nordic walking significantly reduced HbA1c levels in individuals with prediabetes or diabetes compared with the control (Figure 3a; MD = −0.37*%*, 95% CI = −0.56 to −0.18, *I* ^2^ = 69*%*, *p* = 0.0001, power = 97.1*%*). The decreased HbA1c with the Nordic walking, represented by a higher heterogeneity, indicates the involvement of confounding factors like age, baseline HbA1c, intervention duration, and body composition that could affect the outcome.

Figure 3Forest plot illustrating the efficacy of Nordic walking on glycemic control in adults with prediabetes or Type 2 diabetes. (a) glycated hemoglobin (HbA1c); (b) fasting blood glucose (FBG); (c) 2‐hour glucose; and (d) homeostasis model assessment of insulin resistance (HOMA‐IR). SD, standard deviation; CI, confidence interval; IGT, impaired glucose tolerance; T2D, Type 2 diabetes.

**Figure:**
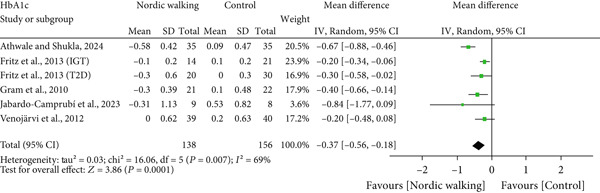
(a)

**Figure:**
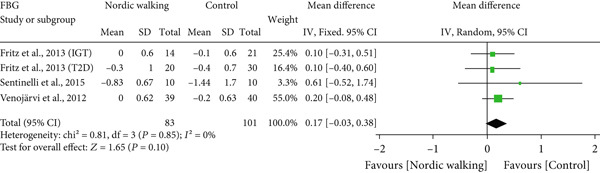
(b)

**Figure:**
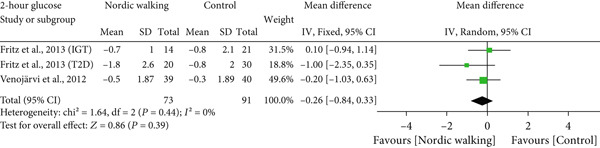
(c)

**Figure:**
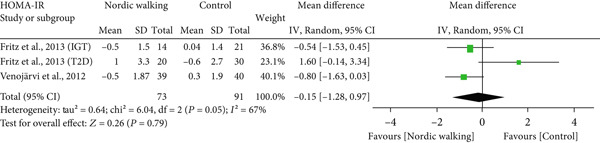
(d)

However, we found no significant differences between Nordic walking and control trials for FBG (Figure 3b; MD = 0.17 mg/dL, 95% CI = −0.03 to 0.38, *I* ^2^ = 0*%*, *p* = 0.10, power = 37.7*%*), 2‐h glucose (Figure 3c; MD = −0.26 mg/dL, 95% CI = −0.84 to 0.33, *I* ^2^ = 0*%*, *p* = 0.39, power = 13.8*%*), and HOMA‐IR (Figure 3d; MD = −0.15, 95% CI = −1.28 to 0.97, *I* ^2^ = 67*%*, *p* = 0.79, power = 5.8*%*). Our findings revealed that Nordic walking is effective in decreasing HbA1c; however, it did not affect blood glucose or HOMA‐IR in participants with prediabetes or diabetes.

#### 3.2.3. Decreased HbA1c is Greater in Diabetes Than Prediabetes After Nordic Walking

**Figure 4:**
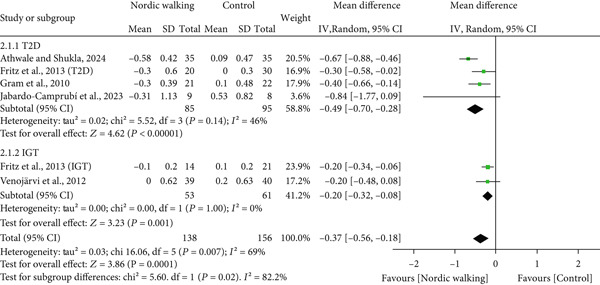
Subgroup analysis of glycated hemoglobin (HbA1c). T2D, Type 2 diabetes; IGT, impaired glucose tolerance; SD, standard deviation; CI, confidence interval.

Based on the HbA1c data, we categorized the trials into two subgroups, prediabetes and diabetes, and subgroup analysis was performed to determine the differential effects of Nordic walking on HbA1c. As shown in Figure 4, two trials comprising 53 individuals with prediabetes represented a significant reduction of HbA1c in the Nordic walking trial compared with control (Figure 4; MD = −0.2*%*, 95% CI = −0.32 to −0.08, *p* = 0.001). Next, the results of four trials involving 85 individuals with diabetes demonstrated a greater reduction of HbA1c with Nordic walking compared with control (Figure 4; MD = −0.49*%*, 95% CI = −0.70 to −0.28, *p* < 0.00001). The decreased HbA1c in individuals with diabetes (MD = −0.49*%*) appears to be greater than that of individuals with prediabetes (MD = −0.2*%*). The test for subgroup analysis results also revealed a statistically significant difference between prediabetes and diabetes subgroups (*p* = 0.02).

#### 3.2.4. Effect of Nordic Walking on Lipid Profile

The changes in lipid profile after Nordic walking were reported in four studies [20, 21, 32, 34], and the differences were compared with the control trial (Figure 5). Here, we emphasize that the Nordic walking intervention duration in all four studies was 12 weeks. Accordingly, the meta‐analysis results showed that the given duration of Nordic walking had no significant effects on TC (Figure 5a; MD = −0.07 *m* *m* *o* *l*/*L*, 95% CI = −0.32 to 0.18, *I* ^2^ = 67*%*, *p* = 0.58, power = 8.6*%*), TG (Figure 5b; MD = −0.11, 95% CI = −0.24 to 0.02, *I* ^2^ = 0*%*, *p* = 0.10, power = 37.8*%*), and LDL cholesterol levels (Figure 5c; MD = −0.10 mmol/L, 95% CI = −0.29 to 0.08, *I* ^2^ = 58*%*, *p* = 0.26, power = 20.3*%*) in patients with prediabetes or diabetes. However, the same duration of Nordic walking had a positive effect on HDL cholesterol, as we found significantly increased HDL levels (Figure 5d; MD = 0.07 mmol/L, 95% CI = 0.02 to 0.12, *I* ^2^ = 0*%*, *p* = 0.005, power = 79.6*%*) in the participants.

Figure 5Forest plot demonstrating the efficacy of Nordic walking on lipid profile in adults with prediabetes or Type 2 diabetes. (a) total cholesterol (TC); (b) triglycerides (TG); (c) low‐density lipoprotein (LDL); and (d) high‐density lipoprotein (HDL). SD, standard deviation; CI, confidence interval; IGT, impaired glucose tolerance; T2D, Type 2 diabetes.

**Figure:**
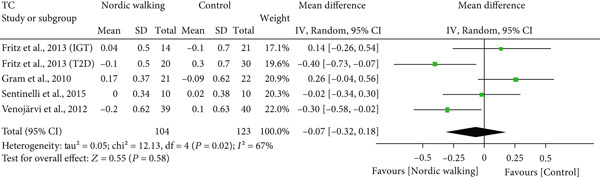
(a)

**Figure:**
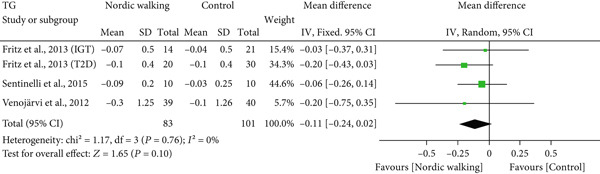
(b)

**Figure:**
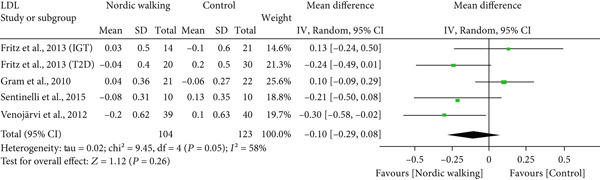
(c)

**Figure:**
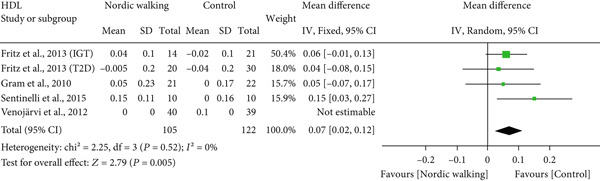
(d)

#### 3.2.5. Effect of Nordic Walking on Blood Pressure and VO_2_peak

Three studies reported changes in blood pressure with Nordic walking and compared them to a control group [20, 21, 32]. From our meta‐analysis results, we found no significant change in either SBP (Figure S2a; MD = 0.34 mmHg, 95% CI = −2.99 to 3.66, *I* ^2^ = 40*%*, *p* = 0.84, power = 5.5*%*) or diastolic blood pressure (Figure S2b; MD = 0.17 mmHg, 95% CI = −1.67 to 2.01, *I* ^2^ = 0*%*, *p* = 0.86, power = 5.4*%*) of participants with prediabetes or diabetes.

**Figure 6:**
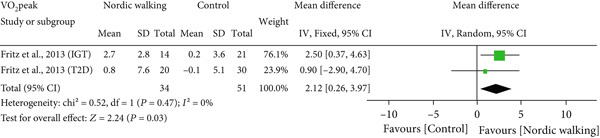
Forest plot demonstrating the effect of Nordic walking on peak oxygen uptake (VO_2_peak) in participants with prediabetes or Type 2 diabetes. SD, standard deviation; CI, confidence interval; IGT, impaired glucose tolerance; T2D, Type 2 diabetes.

Of the six included studies, only one study with two trials reported VO_2_peak data of participants [20]. Our meta‐analysis found that Nordic walking intervention significantly increased peak oxygen uptake (Figure 6; MD = 2.12, 95% CI = 0.26–3.97, *I* ^2^ = 0*%*; *p* = 0.03, power = 60.9*%*) in participants with prediabetes or diabetes compared to the control.

### 3.3. Sensitivity Analysis

Sensitivity analysis was conducted using the “leave‐one study‐out” method to assess the robustness of the pooled effects for all outcomes. Upon excluding the study by Venojärvi et al. [21], the significantly reduced body weight in the Nordic walking trial compared with the control became nonsignificant, suggesting the strong influence of this study. Removing a trial arm involving adults with prediabetes from the Fritz et al. study [20] resulted in a significant reduction in WC (*p* = 0.04), whilst removing the arm with diabetes from the same study [20] led to a significant reduction in HOMA‐IR (*p* = 0.03), which differed from the original pooled effect, suggesting the influence of baseline health status on these outcomes. Similarly, excluding the Gram et al. study [32] from the pooled analysis showed a statistically significant reduction in LDL cholesterol (*p* = 0.009). However, the pooled results for HbA1c, HDL cholesterol, and blood pressure remained robust and were unaffected by sensitivity analyses (Figures S3, S4, S5 and S6).

## 4. Discussion

**Figure 7:**
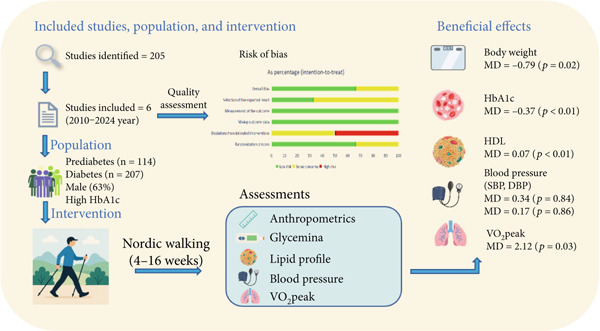
Nordic walking decreased body weight/HbA1c and improved HDL/VO_2_peak in adults with prediabetes or diabetes.

Although previous reviews have indicated the beneficial effects of Nordic walking in populations with CVDs, respiratory diseases and Parkinson′s disease, its efficacy on clinical outcomes in populations with prediabetes or diabetes has not been systematically reviewed. This systematic review and meta‐analysis evaluated the effects of Nordic walking on anthropometrics, glycemic control, lipid profiles, blood pressure, and VO_2_peak in adults with prediabetes or diabetes. To the best of our knowledge, this is the first study to synthesize the evidence using both qualitative and quantitative methodologies. Meta‐analysis results of six RCTs showed that Nordic walking significantly decreased body weight and HbA1c and increased HDL cholesterol and VO_2_peak in participants with prediabetes or diabetes compared with controls. Through the subgroup analysis, we found that the decrease in HbA1c in people with diabetes is greater than that of people with prediabetes. According to the pooled analysis, Nordic walking tends to decrease WC, but does not affect BMI, FBG, 2‐h glucose, and HOMA‐IR. In addition, Nordic walking appeared ineffective in influencing other cardiovascular risk factors, such as TC, TG, LDL, and blood pressure in the studied population. Overall, Nordic walking with a duration of 4‐ to 16‐week can promote weight loss and decrease HbA1c in people with diabetes or prediabetes (Figure 7).

We found that the Nordic walking intervention led to a significant reduction in body weight and decreased tendency of WC compared with the control trial in participants with prediabetes or diabetes, whereas BMI remained unchanged. According to recent findings from RCTs, Nordic walking appears to be ineffective in decreasing BMI and WC in people with diabetes [22, 33]. Contrary, another RCT with 12‐week structured Nordic walking training reported decreased body weight and BMI in women with diabetes [34]. Our findings are consistent with a recent meta‐analysis of 22 RCTs, which reported the decreased body weight after Nordic walking in the elderly, aged 62 years [24]. In contrast, an earlier meta‐analysis of eight RCTs in 2020 concluded that Nordic walking, compared with control, did not affect the body weight and BMI of adults with overweight and obesity [28]. However, this meta‐analysis reported limitations of diverse health statuses of participants in some RCTs and a smaller sample size (*n* = 415) [28]. The duration of Nordic walking in our study and in the above‐mentioned two meta‐analyses ranged from 4‐ to 16‐week [24, 28]; however, the differential effect of Nordic walking on body weight and BMI may be attributed to the differences in health status of the participants. Studies have shown that weight loss in people with diabetes is strongly associated with improvement of diabetic complications [51, 52]. The decreased body weight after Nordic walking in middle‐aged men with prediabetes is associated with reduced adipose tissue mass and body fat percentage [21]. In addition, weight loss, including fat mass reduction, after exercise can help improve insulin sensitivity, hyperglycemia, or blood pressure in people with obesity or diabetes [53–55]. Although statistically significant weight loss was observed in our study, the magnitude is moderate, and the calculated percentage of weight loss did not exceed 3% across the included trials. This outcome suggests that the observed weight loss may not have produced a clinically significant benefit in the studied population. Existing evidence indicates that cardiovascular or metabolic benefits from weight loss interventions can be achieved with a weight loss of > 3% for improving glycemic control and TG, and at least 5% for improving blood pressure, HDL, and LDL cholesterol [56–59]. Nevertheless, Nordic walking remains a practical lifestyle intervention that could lead to substantial weight loss when combined with other strategies, such as dietary modification.

Patients with diabetes are advised to maintain a healthy lifestyle and keep their HbA1c levels below 7.0% to mitigate the disease‐associated complications [60, 61]. A noteworthy finding from our meta‐analysis is that Nordic walking substantially decreased HbA1c without significantly affecting short‐term glycemic markers, like FBG, 2‐h glucose, or HOMA‐IR. Although our findings suggest a unique beneficial effect of Nordic walking on long‐term glycemic control, the existing literature is inconsistent. For example, Venojärvi et al. reported that 12‐week Nordic walking (supervised) had no effect on HbA1c and blood glucose in overweight and obese men with prediabetes [21]. Similarly, a 16‐week Nordic walking (unsupervised) reported unchanged HbA1c in overweight/obese adults with prediabetes; however, decreased HbA1c was reported in overweight and obese adults with diabetes [20]. A recent RCT by Athwale and Shukla demonstrated that 4‐week Nordic walking (supervised) significantly reduced HbA1c in adults with diabetes [31]. These inconsistencies may be due to differences in baseline HbA1c levels and/or the health status of the participants. Regarding the results of meta‐analyses, one study included eight RCTs involving overweight and obese adults [28], and another recent study included 22 RCTs involving older adults [24], and both reported no significant decrease in HbA1c with Nordic walking compared with the control. The evidence from these two meta‐analyses appears consistent with unchanged HbA1c after Nordic walking, but it is not fully supported because the studied population is diverse. It is worth noting that no meta‐analysis synthesized the evidence to demonstrate the effects of Nordic walking on HbA1c in adults exclusively with prediabetes or diabetes.

HbA1c reflects integrated glycemic exposure over a 2‐ to 3‐month period, providing a stable and robust assessment of overall glycemic control. In contrast, FBG and HOMA‐IR are snapshot metrics susceptible to high daily variability from factors like recent diet or acute stress, which may obscure the true underlying effect of a chronic exercise intervention in a meta‐analysis context [62, 63]. Therefore, the significant reduction in HbA1c should be viewed as the primary indicator of the sustained metabolic benefits conferred by Nordic walking. In addition, we explored the efficacy of Nordic walking on HbA1c in people with different baseline HbA1c levels who were referred to prediabetes or diabetes. Subgroup analysis revealed that the decrease in HbA1c with Nordic walking is greater in the diabetes than in the prediabetes subgroup. This suggests a pronounced therapeutic response in individuals with poorer baseline glycemic control and thus a greater capacity for improvement following Nordic walking. This phenomenon is consistent with other exercise interventions, witnessing a greater improvement of glycemic control in patients with higher HbA1c or a longer history of diabetes [64, 65]. For patients with Type 2 diabetes, whose glucose metabolism pathways are impaired, the increased whole‐body muscle engagement and energy expenditure with Nordic walking [66], and increased GLUT4 protein or glycogen synthase activity with aerobic exercise [67], perhaps contributed to restoring glucose metabolism. Given the strong association between lower HbA1c levels and reduced cardiovascular events, the magnitude of HbA1c decrease (−0.49%, *p* = 0.00001) in patients with diabetes may contribute to cardiovascular benefits. Previous evidence reveals that each 1% reduction in HbA1c is associated with a 21% decrease in diabetes‐related deaths, a 37% decrease in microvascular complications, and a 14% reduction in myocardial infarction [68]. Achieving an HbA1c percentage below 7.0% in patients with diabetes leads to an absolute risk reduction of developing coronary heart disease (5%–10%) and all‐cause mortality [69]. Although the magnitude of HbA1c reduction in our study is moderate, Nordic walking remains effective in reducing HbA1c, particularly in adults with higher baseline values. Further studies with a large sample size are necessary to determine the optimal frequency, intensity, or duration of Nordic walking based on participants′ physical fitness and baseline HbA1c levels.

The “Standards of Care in Diabetes” by the American Diabetes Association (ADA) recommends that improving lipid profiles in diabetes through lifestyle modifications can reduce the risk of atherosclerotic cardiovascular disease (ASCVD) [70]. In our meta‐analysis, we found that Nordic walking significantly increased HDL in individuals with prediabetes or diabetes, whereas there were no significant changes in other lipid parameters, including TC, TG, and LDL. This observation is consistent with existing reports that showed HDL cholesterol is more responsive to aerobic exercise than TC, TG, or LDL in adults [71, 72]. An RCT conducted on individuals with obesity reported an increase in HDL cholesterol with aerobic exercise; however, TC, TG, and LDL cholesterol remained unchanged [73]. The unchanged non‐HDL parameters in our analysis may be associated with the differences in intensity or duration of the Nordic walking intervention. It has been suggested that a significant reduction in LDL and TG with exercise often necessitates a greater stimulus by a higher volume or higher intensity [74–76]. Vigorous physical activity with a higher frequency (3–6 times/week) increased HDL and decreased TG in adults, while a lower frequency (1–2 times/week) showed no effect on lipid profile [77]. Concurrently, a structured training protocol alone did not affect HDL, TC, or TG in male recreational athletes with diabetes. However, the training protocol combined with dietary intervention has been shown to improve HDL cholesterol [78]. The statistically significant increase in HDL observed in adults with prediabetes or diabetes after Nordic walking is likely clinically insignificant. Determining a clinically significant change in HDL is complex, as evidence for cardiovascular benefit from simply raising HDL is mixed [79]. According to the 2019 European Society of Cardiology (ESC) and European Atherosclerosis Society (EAS) guidelines, a clear and reproducible inverse relationship exists between HDL cholesterol and ASCVD risk, but there is no evidence from RCTs to suggest that increasing HDL reduces the risk of cardiovascular events [80]. Furthermore, the cardiovascular protection associated with HDL in the general population may not extend to individuals with metabolic diseases [81]. To optimize reductions in TC and TG, and improvements in HDL, Nordic walking protocols for individuals with prediabetes or diabetes should be personalized/tailored based on their baseline lipid profile.

Regarding blood pressure, our meta‐analysis did not find a statistically significant reduction in individuals with prediabetes or diabetes, although a trend towards a modest improvement was observed. This aligns with the findings of Sanchez‐Lastra et al. in overweight or obese populations [28], but contrasts with another meta‐analysis in older adults, where Nordic walking significantly lowered SBP [24]. Nordic walking for 6 months also decreased SBP but did not affect DBP in older adults with diabetes [22]. This discrepancy may be attributable to differences in population characteristics; for instance, the older adults in the study by Liu and Kim may have had higher baseline blood pressure, allowing for a greater magnitude of reduction [24]. Ultimately, definitive conclusions on the blood pressure‐lowering effects of Nordic walking in the diabetic population will require future trials with larger sample sizes and longer durations. In contrast to the blood pressure results, our analysis revealed a significant improvement in VO_2_peak after Nordic walking. This finding is critical, as VO_2_peak is a powerful and integrative measure of cardiorespiratory fitness, reflecting the coordinated efficiency of the cardiovascular, respiratory, and muscular systems [82]. Our results are supported by the evidence from RCT showing that 12‐week Nordic walking training can improve aerobic capacity by approximately 20% in middle‐aged men with prediabetes [21]. Similarly, another RCT with a 4‐week Nordic walking program has shown increased maximal aerobic capacity in patients with diabetes; however, the findings are limited by the shorter intervention duration [31]. Given that each one metabolic equivalent (≈3.5 mL/kg/min) increase in VO_2_peak is associated with a substantial reduction in CVD risk [82, 83], the improvement in VO_2_peak with Nordic walking may represent a profound health benefit for individuals with prediabetes or diabetes.

## 5. Limitations

Although our systematic review and meta‐analysis provide valuable insights into the beneficial effects of Nordic walking, we have a few limitations that should be acknowledged to guide future research. First, the limited number of RCTs and their small sample sizes resulted in insufficient statistical power for most outcomes, and therefore, the results should be interpreted cautiously. For instance, Nordic walking improves VO_2_peak in adults with prediabetes or diabetes; however, this finding was based on only two trials from a single study, which limits robustness and generalization. Due to the small number of included RCTs, we are unable to formally assess publication bias using the funnel plots and Egger′s test. Second, methodological variations across RCTs may have influenced the results. One RCT employed an unsupervised Nordic walking protocol that relied on self‐reported exercise intensity, which raises concerns about adherence accuracy, and its effect on outcomes, especially when compared with the supervised protocols used in other studies. Furthermore, variations in assessment methodologies for glycemic control, lipid profile, and blood pressure across RCTs may represent additional confounding factors that could influence the data. Finally, most participants in our analysis (5 of 6 RCTs) are from Europe; therefore, our findings may not be generalizable to populations from other regions. Given the potential differences in genetic backgrounds, dietary habits, and lifestyles across ethnicities, the effects of Nordic walking on clinical outcomes may differ in other populations. Further studies are necessary to confirm the efficiency of Nordic walking on populations from a wider range of regions or ethnicities.

## 6. Conclusion

Our findings demonstrated the beneficial effects of Nordic walking intervention on glycemic control (particularly on HbA1c) and weight management in adults with prediabetes or diabetes. These findings underscore the clinical significance of incorporating Nordic walking as an effective lifestyle intervention for the prevention and management of diabetes. However, further studies are necessary to explore the precise physiological adaptations to Nordic walking and how these adaptations could promote anthropometrics, glycemia, and lipid profile in adults with prediabetes or diabetes.

## Disclosure

All authors thoroughly reviewed and approved the final version of the manuscript.

## Conflicts of Interest

The authors declare no conflicts of interest.

## Author Contributions

Sichao Chen, Weibing Ye, and Mallikarjuna Korivi designed the study. Sichao Chen, Xiaohong An, Ankang Wu, and Yubo Liu performed the literature search and study selection. Sichao Chen, Xiaohong An, and Ankang Wu extracted data, performed statistical analyses, and drafted the original manuscript. Veeranjaneya Reddy Lebaka and Bhaskar LVKS provided additional insights into the data and manuscript. Mallikarjuna Korivi, Weibing Ye, and Yubo Liu reviewed the data and results. Mallikarjuna Korivi and Weibing Ye revised and finalized the manuscript.

## Funding

This study was supported by Zhejiang Province Maker Space Innovation and Entrepreneurship Training Project: FiveAspect Intelligent Training: Technology‐Empowered Running Service.

## Supporting Information

Additional supporting information can be found online in the Supporting Information section.

## Supporting information


**Supporting Information 1** Table S1: PRISMA checklist.

**Supporting Information 2** Table S2: Detailed search strategy on electronic databases. Table S3: Percentage of weight loss in each study. NR, not reported; IGT, impaired glucose tolerance; T2D, Type 2 diabetes. Figure S1: Risk of bias (RoB2) assessment for included studies. Figure S2: Forest plot demonstrating the efficacy of Nordic walking on blood pressure in adults with prediabetes or Type 2 diabetes. (a) systolic blood pressure (SBP); and (b) diastolic blood pressure (DBP). SD, standard deviation; CI, confidence interval; IGT, impaired glucose tolerance; T2D, Type 2 diabetes. Figure S3 Sensitivity analysis for body composition. BMI, body mass index; WC, waist circumference; MD, mean difference; IGT, impaired glucose tolerance; T2D, Type 2 diabetes. Figure S4 Sensitivity analysis for glycemic control. HbA1c, glycated hemoglobin; FBG, fasting blood glucose; MD, mean difference; IGT, impaired glucose tolerance; T2D, Type 2 diabetes. Figure S5 Sensitivity analysis for lipid profile. TC, total cholesterol; TG, triglycerides; LDL, low‐density lipoprotein; HDL, high‐density lipoprotein; MD, mean difference; IGT, impaired glucose tolerance; T2D, Type 2 diabetes. Figure S6 Sensitivity analysis for blood pressure. SBP, systolic blood pressure; DBP, diastolic blood pressure; MD, mean difference; T2D, Type 2 diabetes; IGT, impaired glucose tolerance.

## Data Availability

The datasets generated and/or analyzed used in this study are available from the corresponding authors on reasonable request.
