# Supplementary material for: Effect of Nordic Walking on Anthropometrics, Glycemia, and Lipid Profile in Adults With Prediabetes or Diabetes: A Systematic Review and Meta‐Analysis of Randomized Controlled Trials
Source: J Diabetes Res. 2026 Jan 8;2026:5886930. doi: 10.1155/jdr/5886930 (PMC12782928; doi:10.1155/jdr/5886930)

**Effect of Nordic Walking on** **Anthropometrics, Glycemia and Lipid Profile in Adults with Prediabetes or Diabetes: A Systematic Review and Meta-Analysis of Randomized Controlled Trials**

Sichao Chen^1^, Xiaohong An^2^, Ankang Wu^1^, Yubo Liu^1^, Veeranjaneya Reddy Lebaka^3^, Bhaskar LVKS^4^, Mallikarjuna Korivi^1^*, Weibing Ye^1^*

**Supplementary Table 2** Detailed search strategy on electronic databases.

| **Database** | **Query** | **Results** |
| --- | --- | --- |
| **PubMed** | (((("Nordic Walking"[Mesh]) OR (Walking, Nordic)) OR (Pole Walking)) OR (Walking, Pole)) AND (((((((((((((((((((((((((((((((((((((((((((((((("Diabetes Mellitus, Type 2"[Mesh]) OR (Diabetes Mellitus, Stable)) OR (Stable Diabetes Mellitus)) OR (Diabetes Mellitus, Noninsulin Dependent)) OR (Diabetes Mellitus, Adult-Onset)) OR (Adult-Onset Diabetes Mellitus)) OR (Diabetes Mellitus, Adult Onset)) OR (Diabetes Mellitus, Ketosis-Resistant)) OR (Diabetes Mellitus, Ketosis Resistant)) OR (Ketosis-Resistant Diabetes Mellitus)) OR (Diabetes Mellitus, Non-Insulin-Dependent)) OR (Non-Insulin-Dependent Diabetes Mellitus)) OR (Diabetes Mellitus, Type II)) OR (NIDDM)) OR (Diabetes Mellitus, Maturity-Onset)) OR (Diabetes Mellitus, Maturity Onset)) OR (Maturity-Onset Diabetes Mellitus)) OR (Maturity Onset Diabetes Mellitus)) OR (MODY)) OR (Diabetes Mellitus, Slow-Onset)) OR (Diabetes Mellitus, Slow Onset)) OR (Slow-Onset Diabetes Mellitus)) OR (Type 2 Diabetes Mellitus)) OR (Noninsulin-Dependent Diabetes Mellitus)) OR (Noninsulin Dependent Diabetes Mellitus)) OR (Maturity-Onset Diabetes)) OR (Diabetes, Maturity-Onset)) OR (Maturity Onset Diabetes)) OR (T2DM)) OR (Type 2 Diabetes)) OR (Diabetes, Type 2)) OR (Diabetes Mellitus, Noninsulin-Dependent)) OR (Insulin Resistance)) OR (Resistance, Insulin)) OR (Insulin Sensitivity)) OR (Sensitivity, Insulin)) OR (Glucose Intolerance)) OR (Glucose Intolerances)) OR (Glucose Intolerances)) OR (Intolerances, Glucose)) OR (Impaired Glucose Tolerance)) OR (Glucose Tolerance, Impaired)) OR (Glucose Tolerances, Impaired)) OR (Impaired Glucose Tolerances)) OR (Tolerance, Impaired Glucose)) OR (Tolerances, Impaired Glucose)) OR (homeostasis model assessment of insulin resistance)) OR (HOMA‑IR)) | 32 |
| Web of Science | (TS=(Nordic Walking) OR AB=(Walking, Nordic OR Pole Walking OR Walking, Pole)) AND (TS=(Diabetes Mellitus, Type 2) OR AB=(Diabetes Mellitus, Stable OR Stable Diabetes Mellitus OR Diabetes Mellitus, Noninsulin Dependent OR Diabetes Mellitus, Adult-Onset OR Adult-Onset Diabetes Mellitus OR Diabetes Mellitus, Adult Onset OR Diabetes Mellitus, Ketosis-Resistant OR Diabetes Mellitus, Ketosis Resistant OR Ketosis-Resistant Diabetes Mellitus OR Diabetes Mellitus, Non Insulin Dependent OR Diabetes Mellitus, Non-Insulin-Dependent OR Non-Insulin-Dependent Diabetes **Mellitus OR Diabetes Mellitus, Type II OR NIDDM OR Diabetes Mellitus, Maturity-Onset OR Diabetes Mellitus, Maturity Onset OR Maturity-Onset Diabetes Mellitus OR Maturity Onset Diabetes Mellitus OR MODY OR Diabetes Mellitus, Slow-Onset OR Diabetes Mellitus, Slow Onset OR Slow-Onset Diabetes Mellitus OR Type 2 Diabetes Mellitus OR Noninsulin-Dependent Diabetes Mellitus OR Noninsulin Dependent Diabetes Mellitus OR Maturity-Onset Diabetes OR Diabetes, Maturity-Onset OR Maturity Onset Diabetes OR Type 2 Diabetes OR Diabetes, Type 2 OR T2DM OR Diabetes Mellitus, Noninsulin-Dependent OR Insulin Resistance OR Impaired Glucose Tolerance OR** homeostasis model assessment of insulin resistance **OR HOMA‑IR))** | 69 |
| The Cochrane Library | **#6 MeSH descriptor: [Nordic Walking] explode all trees**  **#11 Nordic Walking or Walking, Nordic or Pole Walking or Walking, Pole**  **#12 #6 or #11**  **#9 MeSH descriptor: [Diabetes Mellitus, type 2] explode all trees**  **#13 Diabetes Mellitus, type 2 or Diabetes Mellitus, Stable or Stable Diabetes Mellitus or Diabetes Mellitus, Noninsulin Dependent or Diabetes Mellitus, Adult-Onset or Adult-Onset Diabetes Mellitus or Diabetes Mellitus, Adult Onset or Diabetes Mellitus, Ketosis-Resistant or Diabetes Mellitus, Ketosis Resistant or Ketosis-Resistant Diabetes Mellitus or Diabetes Mellitus, Non Insulin Dependent or Diabetes Mellitus, Non-Insulin-Dependent or Non-Insulin-Dependent Diabetes Mellitus or Diabetes Mellitus, Type II or NIDDM or Diabetes Mellitus, Maturity-Onset or Diabetes Mellitus, Maturity Onset or Maturity-Onset Diabetes Mellitus or Maturity Onset Diabetes Mellitus or MODY or Diabetes Mellitus, Slow-Onset or Diabetes Mellitus, Slow Onset or Slow-Onset Diabetes Mellitus or Type 2 Diabetes Mellitus or Noninsulin-Dependent Diabetes Mellitus or Noninsulin Dependent Diabetes Mellitus or Maturity-Onset Diabetes or Diabetes, Maturity-Onset or Maturity Onset Diabetes or Type 2 Diabetes or Diabetes, Type 2 or T2DM or Diabetes Mellitus, Noninsulin-Dependent or Impaired Glucose Tolerance or Insulin Resistance or** homeostasis model assessment of insulin resistance **or HOMA‑IR**  **#14 #9 or #13**  **#15 #12 and #14** | 31 |
| Scopus | **( TITLE-ABS-KEY ( Nordic Walking ) OR TITLE-ABS-KEY ( Nordic Walking ) OR TITLE-ABS-KEY ( Walking, Nordic ) OR TITLE-ABS-KEY ( Pole Walking ) OR TITLE-ABS-KEY ( Walking, Pole ) AND TITLE-ABS-KEY ( Diabetes Mellitus, Type 2 ) OR TITLE-ABS-KEY ( Diabetes Mellitus, Stable ) OR TITLE-ABS-KEY ( Stable Diabetes Mellitus ) OR TITLE-ABS-KEY ( Diabetes Mellitus, Noninsulin Dependent ) OR TITLE-ABS-KEY ( Diabetes Mellitus, Adult-Onset ) OR TITLE-ABS-KEY ( Adult-Onset Diabetes Mellitus ) OR TITLE-ABS-KEY ( Diabetes Mellitus, Adult Onset ) OR TITLE-ABS-KEY ( Diabetes Mellitus, Ketosis-Resistant ) OR TITLE-ABS-KEY ( Diabetes Mellitus, Ketosis Resistant ) OR** **TITLE-ABS-KEY ( Ketosis-Resistant Diabetes Mellitus ) OR TITLE-ABS-KEY ( Diabetes Mellitus, Non Insulin Dependent ) OR TITLE-ABS-KEY ( Diabetes Mellitus, Non-Insulin-Dependent ) OR TITLE-ABS-KEY ( Non-Insulin-Dependent Diabetes Mellitus ) OR TITLE-ABS-KEY ( Diabetes Mellitus, Type iii ) OR TITLE-ABS-KEY ( NIDDM ) OR TITLE-ABS-KEY ( Diabetes Mellitus, Maturity-Onset ) OR TITLE-ABS-KEY ( Diabetes Mellitus, Maturity Onset ) OR TITLE-ABS-KEY ( Maturity-Onset Diabetes Mellitus ) OR TITLE-ABS-KEY ( Maturity Onset Diabetes Mellitus ) OR TITLE-ABS-KEY ( MODY ) OR TITLE-ABS-KEY ( Diabetes Mellitus, Slow-Onset ) OR TITLE-ABS-KEY ( Diabetes Mellitus, Slow Onset ) OR TITLE-ABS-KEY ( Slow-Onset Diabetes Mellitus ) OR TITLE-ABS-KEY ( Type 2 Diabetes Mellitus ) OR TITLE-ABS-KEY ( Noninsulin-Dependent Diabetes Mellitus ) OR TITLE-ABS-KEY ( Noninsulin Dependent Diabetes Mellitus ) OR TITLE-ABS-KEY ( Maturity-Onset Diabetes ) OR TITLE-ABS-KEY ( Diabetes, Maturity-Onset ) OR TITLE-ABS-KEY ( Maturity Onset Diabetes ) OR TITLE-ABS-KEY ( Type 2 Diabetes ) OR TITLE-ABS-KEY ( Diabetes, Type 2 ) OR TITLE-ABS-KEY ( T2DM ) OR TITLE-ABS-KEY ( Diabetes Mellitus, Noninsulin-Dependent ) OR TITLE-ABS-KEY ( Impaired Glucose Tolerance ) OR TITLE-ABS-KEY ( Insulin Resistance ) OR TITLE-ABS-KEY (** homeostasis model assessment of insulin resistance **) OR TITLE-ABS-KEY ( HOMA‑IR ))** | 36 |
| Embase | **#1 'nordic walking'/exp OR 'nordic walking' OR 'walking, nordic' OR 'pole walking'/exp OR 'pole walking' OR 'walking, pole'**  **#2 'diabetes mellitus, type' OR 'diabetes mellitus, stable' OR 'stable diabetes mellitus' OR 'diabetes mellitus, noninsulin dependent' OR 'diabetes mellitus, adult-onset' OR 'adult-onset diabetes mellitus'/exp OR 'adult-onset diabetes mellitus' OR 'diabetes mellitus, adult onset' OR 'diabetes mellitus, ketosis-resistant' OR 'diabetes mellitus, ketosis resistant' OR 'ketosis-resistant diabetes mellitus'/exp OR 'ketosis-resistant diabetes mellitus' OR 'diabetes mellitus, non insulin dependent'/exp OR 'diabetes mellitus, non insulin dependent' OR 'diabetes mellitus, non-insulin-dependent'/exp OR 'diabetes mellitus, non-insulin-dependent' OR 'non-insulin-dependent diabetes mellitus'/exp OR 'non-insulin-dependent diabetes mellitus' OR 'diabetes mellitus, type ii'/exp OR 'diabetes mellitus, type ii' OR 'niddm'/exp OR 'niddm' OR 'diabetes mellitus, maturity-onset'/exp OR 'diabetes mellitus, maturity-onset' OR 'diabetes mellitus, maturity onset'/exp OR 'diabetes mellitus, maturity onset' OR 'maturity-onset diabetes mellitus'/exp OR 'maturity-onset diabetes mellitus' OR 'maturity onset diabetes mellitus'/exp OR 'maturity onset diabetes mellitus' OR 'mody' OR 'diabetes mellitus, slow-onset' OR 'diabetes mellitus, slow onset' OR 'slow-onset diabetes mellitus' OR 'type 2 diabetes mellitus'/exp OR 'type 2 diabetes mellitus' OR 'noninsulin-dependent diabetes mellitus'/exp OR 'noninsulin-dependent diabetes mellitus' OR 'noninsulin dependent diabetes mellitus'/exp OR 'noninsulin dependent diabetes mellitus' OR 'maturity-onset diabetes'/exp OR 'maturity-onset diabetes' OR 'diabetes, maturity-onset' OR 'maturity onset diabetes'/exp OR 'maturity onset diabetes' OR 'type 2 diabetes'/exp OR 'type 2 diabetes' OR 'diabetes, type 2'/exp OR 'diabetes, type 2' OR 'diabetes mellitus, noninsulin-dependent' OR 'Impaired Glucose Tolerance' OR 'Insulin Resistance' OR '**homeostasis model assessment of insulin resistance**' OR 'HOMA‑IR'**  #1 AND #2 | 37 |

**Supplementary Table 3.** Percentage of weight loss in the included studies.

| **Study details** | **Baseline body weight (kg)**  **Mean ± SD** | **Percentage of weight loss (%)** |
| --- | --- | --- |
| Athwale and Shukla, 2024 | NR | NR |
| Fritz *et al.*, 2013 | IGT: 92.5±14.7  T2D: 91.9±13.1 | IGT: 0.5  T2D: 1.1 |
| Gram *et al.*, 2010 | T2D: 88.9±14.3 | T2D: 1.8 |
| Jabardo-Camprubí *et* *al.*, 2023 | NR | NR |
| Sentinelli *et al.*, 2015 | T2D: 82.5±16.1 | T2D: 2.9 |
| Venojärvi *et al.*, 2012 | IGT: 95.1±11.7 | IGT: 2.4 |

**Supplementary Fig. 1** Risk of bias (RoB 2) assessment for included studies.


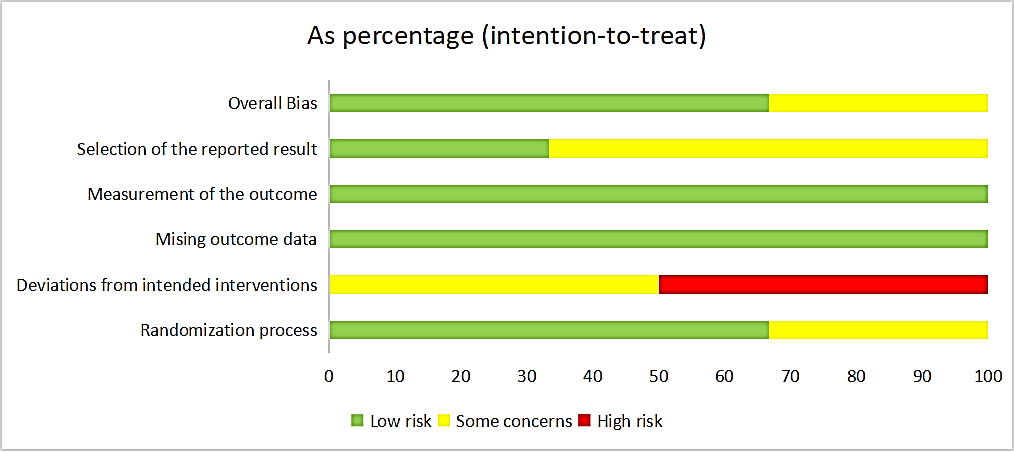

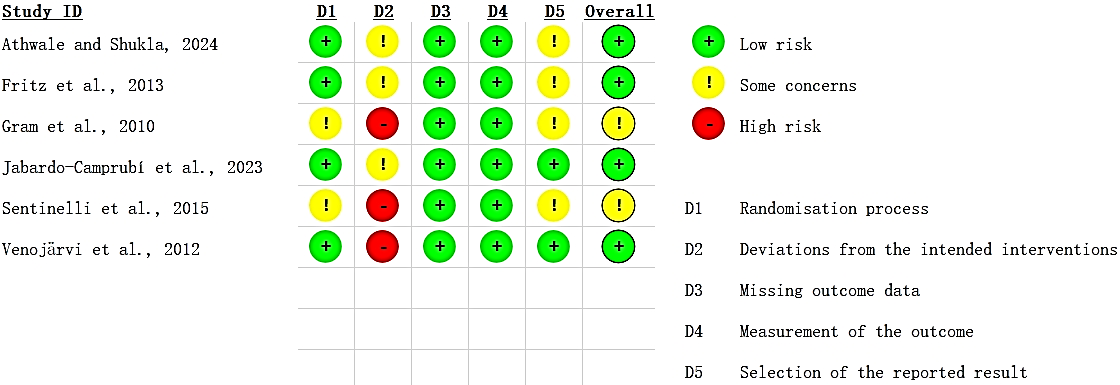


**Supplementary Fig. 2** Forest plot demonstrating the efficacy of Nordic walking on blood pressure in adults with prediabetes or type 2 diabetes. (A) systolic blood pressure (SBP); and (B) diastolic blood pressure (DBP). SD, standard deviation; CI, confidence interval; IGT, impaired glucose tolerance; T2D, type 2 diabetes.


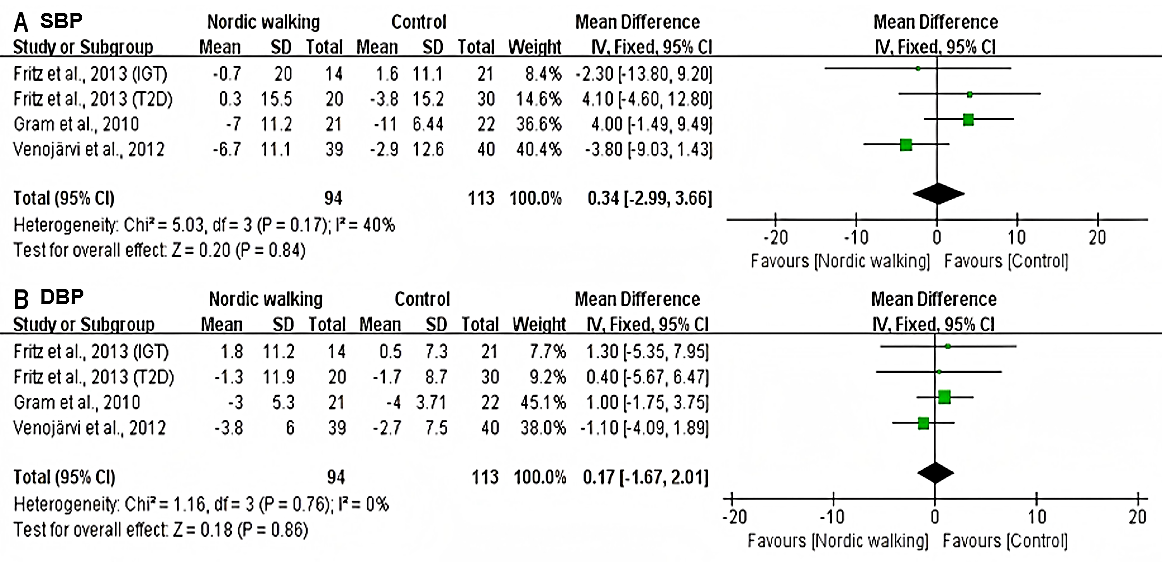


**Supplementary Fig. 3** Sensitivity analysis for body composition. BMI, body mass index; WC, waist circumference; MD, mean difference; IGT, impaired glucose tolerance; T2D, type 2 diabetes.


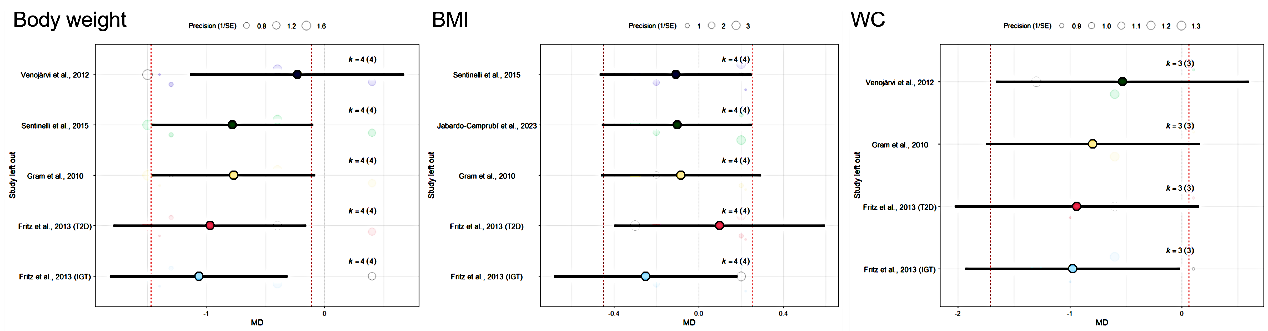


**Supplementary Fig. 4** Sensitivity analysis for glycemic control. HbA1c, glycated hemoglobin; FBG, fasting blood glucose; MD, mean difference; IGT, impaired glucose tolerance; T2D, type 2 diabetes.


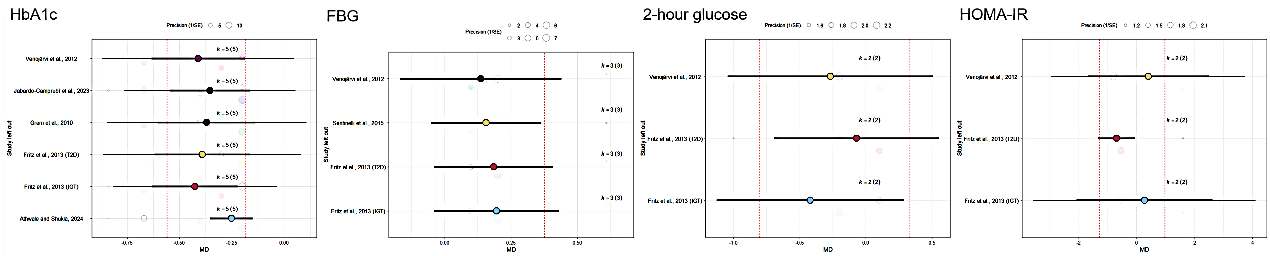


**Supplementary Fig. 5** Sensitivity analysis for lipid profile. TC, total cholesterol; TG, triglycerides; LDL, low-density lipoprotein; HDL, high-density lipoprotein; MD, mean difference; IGT, impaired glucose tolerance; T2D, type 2 diabetes.


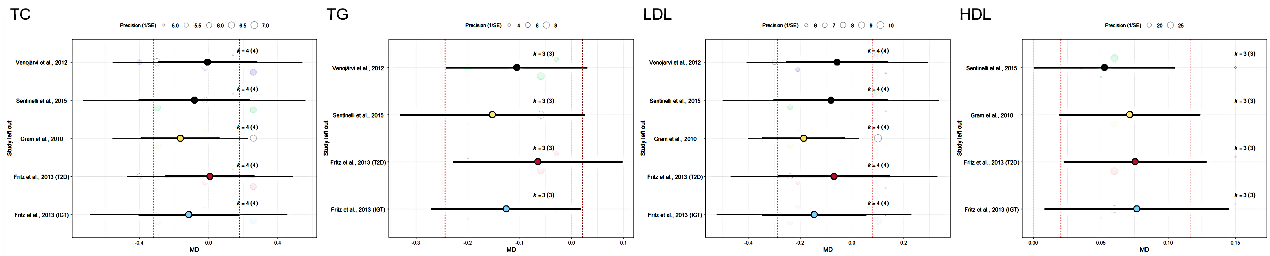


**Supplementary Fig. 6** Sensitivity analysis for blood pressure. SBP, systolic blood pressure; DBP, diastolic blood pressure; MD, mean difference; T2D, type 2 diabetes; IGT, impaired glucose tolerance.


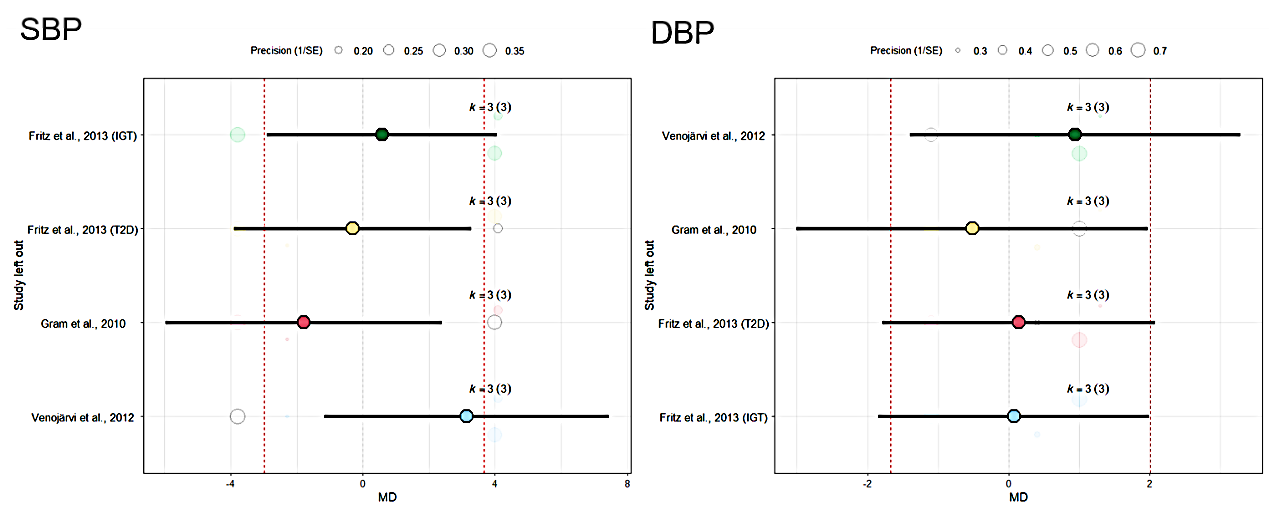

Supplement: Supplementary file 2 — Supporting Information 2 Table S2: Detailed search strategy on electronic databases. Table S3: Percentage of weight loss in each study. NR, not reported; IGT, impaired glucose tolerance; T2D, Type 2 diabetes. Figure S1: Risk of bias (RoB2) assessment for included studies. Figure S2: Forest plot demonstrating the efficacy of Nordic walking on blood pressure in adults with prediabetes or Type 2 diabetes. (a) systolic blood pressure (SBP); and (b) diastolic blood pressure (DBP). SD, standard deviation; CI, confidence interval; IGT, impaired glucose tolerance; T2D, Type 2 diabetes. Figure S3 Sensitivity analysis for body composition. BMI, body mass index; WC, waist circumference; MD, mean difference; IGT, impaired glucose tolerance; T2D, Type 2 diabetes. Figure S4 Sensitivity analysis for glycemic control. HbA1c, glycated hemoglobin; FBG, fasting blood glucose; MD, mean difference; IGT, impaired glucose tolerance; T2D, Type 2 diabetes. Figure S5 Sensitivity analysis for lipid profile. TC, total cholesterol; TG, triglycerides; LDL, low‐density lipoprotein; HDL, high‐density lipoprotein; MD, mean difference; IGT, impaired glucose tolerance; T2D, Type 2 diabetes. Figure S6 Sensitivity analysis for blood pressure. SBP, systolic blood pressure; DBP, diastolic blood pressure; MD, mean difference; T2D, Type 2 diabetes; IGT, impaired glucose tolerance. [file JDR-2026-5886930-s001.docx]
